# The effect of high intensity interval training on cardioprotection against ischemia-reperfusion injury in wistar rats

**DOI:** 10.17179/excli2014-587

**Published:** 2015-02-20

**Authors:** Mostafa Rahimi, Shahnaz Shekarforoush, Ali Reza Asgari, Ali Khoshbaten, Hamid Rajabi, Behzad Bazgir, Mohammad Taghi Mohammadi, Vahid Sobhani, Abolfazl Shakibaee

**Affiliations:** 1 Exercise Physiology Research Center, Baqiyatallah University of Medical Sciences, Tehran, Iran; 2 Department of Physiology, Arsanjan Branch, Islamic Azad University, Fars, Iran; 3 Faculty of Aerospace Medicine, AJA University of Medicine Sciences, Tehran, Iran; 4 Faculty of Physical Education and Exercise Sciences, Kharazmi University, Tehran, Iran; 5 Department of Physiology and Biophysics, Faculty of Medicine, Baqiyatallah University of Medical Sciences, Tehran, Iran

**Keywords:** ischemia, reperfusion, cardioprotection, exercise training

## Abstract

The aims of the present study were to determine whether short term high intensity interval training (HIIT) could protect the heart against ischemia reperfusion (IR) injury; and if so, to evaluate how long the exercise-associated protection can be lasted. Sixty-three rats were randomly assigned into sedentary (n = 15), sham (n = 7), and exercise groups (n = 41). Rats in the exercise groups performed 5 consecutive days of HIIT on treadmill: 5 min warm up with 50 % VO_2_max, 6×2 min with 95-105 % VO_2_max (about 40 to 45 m/min), 5×2 min recovery with 65-75 % VO_2_max (about 28 to 32 m/min), and 3 min cool down with 50 % VO_2_max, all at 0 % grade. Animals exposed to an *in vivo* cardiac IR surgery, performed at days 1, 7, and 14 following the final exercise session. Ischemia-induced arrhythmias, myocardial infarct size (IS), plasma lactate dehydrogenase (LDH) and creatine kinase (CK) activities were measured in all animals. Compared to sedentary rats, exercised animals sustained less IR injury as evidenced by a lower size of infarction and lower levels of LDH and CK at day one and day 7 post exercise. In comparison of sedentary group, IS significantly decreased in EX-IR1 and EX-IR7 groups (50 and 35 %, respectively), but not in EX-IR14 group (19 %). The exercise-induced cardioprotection disappeared 14 days following exercise cessation. There were no significant changes in ischemia-induced arrhythmia between exercised and sedentary rats. The results clearly demonstrate that HIIT protects the heart against myocardial IR injury. This protective effect can be sustained for at least one week following the cessation of the training.

## Introduction

Cardiovascular diseases resulting in ischemic heart disease remain a major cause of morbidity and mortality all over the world. Hence, developing a reasonable and practical countermeasure to provide cardioprotection against ischemia reperfusion (IR) injury is essential. Over the years, investigators have studied several procedures to protect the heart against IR injury. One of the most potent stimuli for eliciting tolerance to IR injury is exercise (Quindry and Hamilton, 2007[26]). Calvert and Lefer (2013[5]) at a review of literature showed that following of all exercise types, cardiac cell death has been reduced by 4 to 75 % and concluded that exercise training reduced infarct size by on average 34 % in animal models (Calvert and Lefer, 2013[5]). Strong body of evidence state that continuous endurance training (CET) protects the heart against IR (Powers et al., 2008[24]; Demirel et al., 2001[7]; Kavazis, 2009[17]). Certainly, in human epidemiological studies it was shown that myocardial-IR induced cell death reduced following regular exercise (Ignarro et al., 2007[15]). Additionally, animal studies have shown that aerobic exercise (both short-term and long-term CET) confers cardioprotection against all levels of IR-induced injury (Frasier et al., 2011[8]; Lennon et al., 2004[18], [19]; Quindry et al., 2005[25]; Calvert, 2011[3]; Powers et al., 2008[24]; Calvert and Lefer, 2013[5]). Lennon et al. (2004a[18]) concluded that both moderate and high-intensity continuous endurance training brings about same myocardial protection against IR injury. Therefore, it seems that both duration and intensity of exercise are significant factors in accomplishing exercise-induced cardioprotection (EICP) (Kavazis, 2009[17]). However, many investigators report that cardiovascular, muscle, and metabolic adaptations in healthy and patient populations are intensity-dependent and the beneficial effects of high intensity interval training (HIIT) rather than CET are better and more reliable for cardioprotection (Gibala et al., 2012[11], 2006[12]; Haram et al., 2009[14]; Tjønna et al., 2008[35]; Freyssin et al., 2012[9]; Guiraud et al., 2012[13]; Wisløff et al., 2007[39], 2009[37]). 

HIIT is characterized by repeated bouts of high-intensity exercise interspersed by periods of rest or low-intensity exercise for recovery (Gibala and Jones, 2013[10]). According to the results of the previous studies, high-intensity exercise protect the heart against IR-induced diastolic dysfunction (Libonati et al., 2005[20]) and is recommended for patients with coronary heart disease (Rognmo et al., 2012[29]). Moreover, the existing evidence recommended that exercise intensity rather than duration and frequency is the most critical factor determining EICP (Wisløff et al., 2006[38]; Tabata et al., 1996[34]; Swain and Franklin, 2006[32]; Rankin et al., 2012[28]). Some of the studies have shown the advantages of the higher-intensity exercise over the moderate-intensity, in cardioprotection (Rankin et al., 2012[28]). Whereas considerable evidence point out that CET leads to EICP, it is uncertain whether other forms of exercise bring about EICP against IR injury. The question remains to be answered is, what is the best type of exercise in order to gain the paramount cardioprotection against IR injury? 

Doubtlessly, EICP vanishes after the termination of exercise training (Lennon et al., 2004[19]). However, how long would it take for the EICP against IR to disappear once the exercise is terminated? In this context, Lennon et al. (2004[19]) reported that after 3 days of CET (60 min, 30 m/min, ~70 % VO_2_max), protection against myocardial stunning remains up to 9 days and is lost 18 days after exercise termination. Calvert et al. (2011[3]) reported that after 4 weeks of voluntary exercise (~7.4 ± 0.2 km/d), EICP against IR was only retained for a week after detraining. Therefore, we expected that HIIT-induced cardioprotection to be more preserved compared to CET (9 days) and voluntary (1 wk) training.

The purpose of the present study was to investigate whether short-term HIIT could reduce the incidence of ischemic-induced arrhythmias and infarct size; and if so, how long this protective effect is retained after exercise cessation. 

## Materials and Methods

### Animals and experimental design

All experimental procedures were approved by the Animal Ethics Committee of Baqiyatallah University of Medical Sciences (396/1392). 

A total of 63 adult male Wistar rat; (~250–320 g) were randomly assigned into the sedentary (n= 22), and exercise groups (n= 41). Animals in the sedentary group were divided into sham (Sham) and control ischemic rats (CO-IR group). Rats of sham group underwent surgery without inducing ischemia, but in control ischemic rats surgery was done in accompany with ischemia induction. Exercised rats were randomly divided into the following sub-groups: a) Ex + IR injury performed after 1 day of rest (Ex-IR1), b) Ex + IR injury performed after 7 days of rest (Ex-IR7), and c) Ex + IR injury performed after 14 days of rest (Ex-IR14). During the experimental period, all animals were maintained on a 12:12-h light-dark period and provided food and water *ad libitum*.

### Exercise protocol

**Figure 1:**
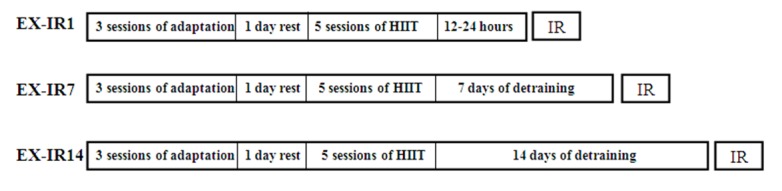
The time line of HIIT training and follow up program

Exercise was carried out for 8 days. The habituation period to the treadmill (3 days) involved a gradual increase in running time, beginning with 10 min of running and ending with 30 min/day at 10-30 m/min, 0 % grade. After 1 day of rest, the animals performed 5 consecutive days of treadmill HIIT exercise: 5 min warm up with 50 % VO_2_max, 6×2 min with 95-105 % VO_2_max (about 40 to 45 m/min), 5×2 min recovery with 65-75 % VO_2_max (about 28 to 32 m/ min), and 3 min cool down with 50 % VO_2_max, all at 0 % grade. Workout intensities were based on preceding understanding of rodent metabolic responses to treadmill running (Lennon et al., 2004[19]; Kavazis, 2009[17]). Rats running poor in two consecutive days were excluded (7 in total). Exercised rats were detrained 1, 7 and 14 days after the last exercise session, and then exposed to IR injury at the selected times (Figure 1(Fig. 1)). IR in EX-IR1 was performed 12-24 h following the final exercise session. 

### IR surgical procedures

Coronary artery ligation and release were performed according to our previous study (Shekarforoush and Foadoddini, 2012[30]). In brief, rats were anesthetized with thiopental sodium (60 mg/kg i.p.). The body temperature was maintained at 37 ± 0.5 °C. A midline incision was made in the neck, a tracheotomy was performed, and rats were artificially ventilated (Harvard Apparatus, South Natica, MA) with room air supplemented with 100 % oxygen. The left carotid artery was cannulated by a catheter for blood pressure monitoring. The electrocardiogram (lead II) was monitored and recorded using subcutaneous needle electrodes and a computerized data acquisition system (ML750 Power Lab/8SP; AD Instruments Pty Ltd, Castle Hill, Australia). After left-sided thoracotomy, the pericardium was incised, a 5-0 silk suture was placed around the left descending coronary artery (LAD) and the ends of it were threaded through a polyethylene tube to form a snare. Rats were then permitted to stabilize for 15 min. Animals with dysrhythmias or a sustained reduction in mean arterial pressure to less than 60 mmHg during the procedure were excluded from the study. For IR injury, the LAD was occluded for 30 min followed by 90 min of reperfusion. Myocardial ischemia was confirmed by ST elevation in the electrocardiogram and a drop in blood pressure.

### Determination of area at risk and infarct size

After IR procedure, the LAD was reoccluded and Evans blue dye (1 ml, 2 %) was injected through right atrium to identify the non-perfused area, as area at risk (AAR). The heart was then removed rapidly; the atria, right ventricle and fatty tissues were removed before the left ventricle was frozen at -20 °C (2 h). Subsequently, the frozen heart was cut into five 2 mm thick sections. An image was taken (Canon Scan Lid 25) from both sides of each slice and all calculations from one heart (using Image Tool Software) were averaged into one value for the purpose of statistical analysis. The area that remained unstained by Evans blue was pale and defined as AAR. Then, all pieces were incubated in 1 % solution of triphenyl-tetrazolium-chloride (TTC) solution in pH 7.4 phosphate buffer for 20 min at 37 °C to visualize the infarct size (IS). Then, slices were fixed for 24 h in 10 % formalin to enhance the contrast. Again, another image was obtained from both sides of each slice. IS, as the white color area, was expressed as a percentage of AAR (IS/AAR×100).

### Evaluation of ischemic-induced arrhythmias

Using the ECG signals, ischemia-induced ventricular arrhythmias were assessed according to the Lambeth conventions (Walker et al., 1988[36]): premature ventricular contraction (PVC) are discrete and identifiable premature QRS complex (premature with respect to the P wave). In addition, bigeminy and salvos were added to PVC count. Ventricular tachycardia (VT) is defined as a run of four or more PVC at a rate faster than the resting sinus rate. Ventricular fibrillation (VF) is defined as a signal where QRS complexes could not easily be distinguished from each other and heart rate could no longer be measured. The number of PVCs, the episodes of VT and the incidence of VF were determined. The duration of VT and VF were also recorded. Rats were excluded from the analysis when either arrhythmias occurred before LAD occlusion (stable period) or no changes in the ST-segment after occlusion the ligature. Additionally, a parametric arrhythmia scoring system was applied to measure ventricular arrhythmia outcomes in accordance with Lambeth Conventions guidelines (Miller et al., 2012[22]). ECG scoring system criteria was in accordance with following formula: 

Score: (log10PVCs) + (log10episodes VT) + 2 [(log10episodes of VF) + (log10 total duration of VF)] (Miller et al., 2012[22]). 

### Measurement of plasma lactate dehydrogenase (LDH) and creatine kinase (CK) activities

At the end of the reperfusion, blood sample (1 ml) was collected and separated serum was stored at 20 °C. Activities of CK and LDH were measured spectrophotometrically (Beckman DU 640, USA) by standard kits (Parsazmoon, Tehran, Iran), and expressed as unit per milliliter (U/mL). 

### Statistical analysis

All values are expressed as means ± SEM. Within each group, data of homodynamic parameters were analyzed using repeated measures analysis of variance. One way analysis of variance (ANOVA) was used to assess an overall difference among the groups for each of the variables. If ANOVA was significant, comparison with the sedentary group was performed by Tukey multiple comparison tests. P = 0.05 was considered to be significant (analysis was performed using SPSS for Windows, version 16).

## Results

### Hemodynamic parameters

The hemodynamic data of this study are summarized in Table 1(Tab. 1). The results showed that no significant differences existed at values of heart rate and mean arterial blood pressure (MBP) among the experimental groups throughout the procedure (p = 0.18, p = 0.29). Compared to pre-ischemic values, MBP was significantly depressed during ischemia and reperfusion in all groups, exception sham.

### Body weight and heart weight

There are significant differences in body weight (p = 0.007) and heart weight (p < 0.001) among groups, but no difference existed in heart- to body weight ratio (p = 0.25) in experimental groups (Table 2(Tab. 2)).

### Area at risk and infarct size

**Figure 2:**
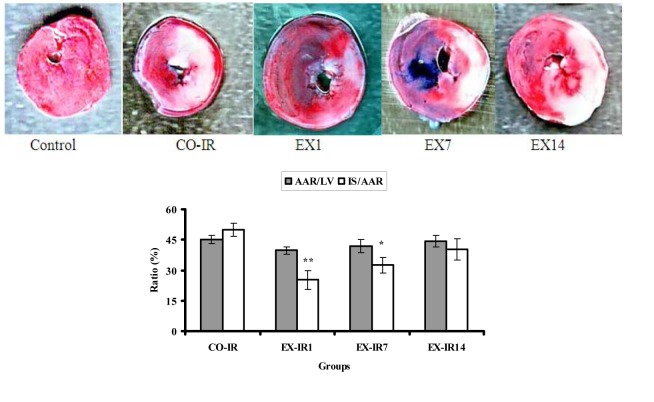
The ratio of myocardial area at risk to left ventricle area (AAR/LV %) and infarct size to area at risk (IS/AAR %) in rats subjected to 30 min ischemia and 90 min of reperfusion. Red areas indicate myocardium within the area at risk for infarction and whitish areas indicate infarcted tissue. CO-IR, control + ischemia–reperfusion; EX-IR1, exercise + IR performed after a day of rest; EX-IR7, exercise + IR performed after 7 days of rest; EX-IR14, exercise + IR performed after 14 days of rest. *P < 0.05 and **P < 0.01 vs CO-IR group

The effects of HIIT on post-exercise infarct size at each time point are presented in Figure 2(Fig. 2). Thirty minutes of ischemia followed by 90 min of reperfusion resulted in a marked myocardial infarction in both sedentary and exercised rats. Exercise was associated with a decrease in infarct area in all exercised groups regardless of detraining time. The result demonstrated that mean of AAR did not differ significantly among groups (p = 0.74), whereas there was significant difference in IS result (p = 0.001). The result of Tukey test indicated that IS was decreased significantly in the EX-IR1 (p = 0.001) and EX-IR7 (p = 0.02) groups, but not in EX-IR14 (p = 0.33), compared with the CO-IR group. In comparision of control group, IS decreased 50, 35, and 19 % in EX-IR1, EX-IR7, and EX-IR14 respectively.

### Ischemia-induced arrhythmias

**Figure 3:**
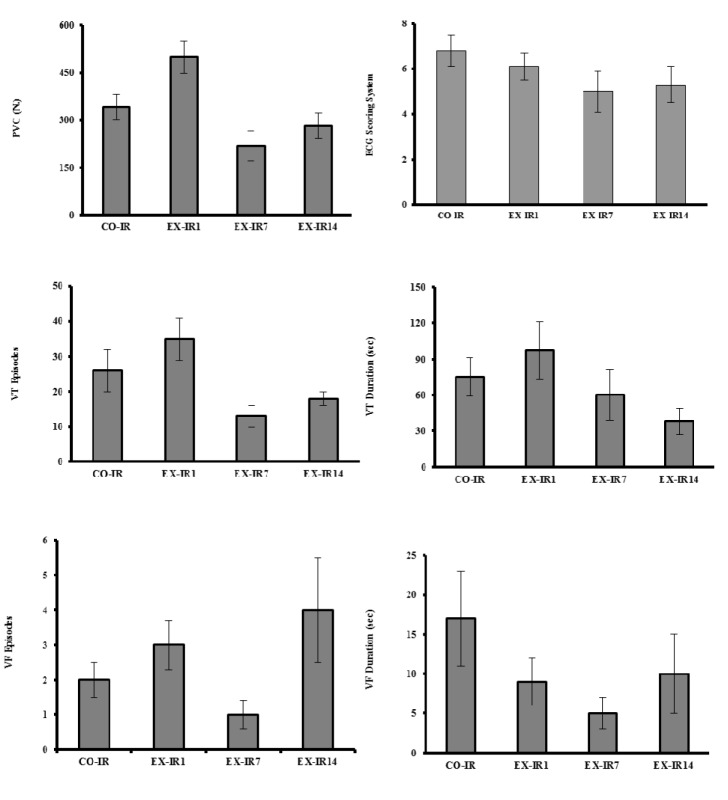
The numbers of premature ventricular complexe (PVC), the episodes of ventricular tachycardia (VT) and ventricular fibrillation (VF), VT and VF duration (as means ± SEM) during 30 min ischemia in rats. CO-IR, control + ischemia–reperfusion; EX-IR1, exercise + IR performed after 1 day of rest; EX-IR7, exercise + IR performed after 7 days of rest; EX-IR14, exercise + IR performed after 14 days of rest

The results of HIIT on ischemia-induced arrhythmias are shown in Figure 3(Fig. 3). During 30 min ischemia, the numbers of PVC, episodes of VT and duration of VT in the CO-IR group were 341 ± 41, 26 ± 6 and 75 ± 16 sec, respectively. Exercise produced no significant reduction in the arrhythmias. There were no considerable reperfusion induced arrhythmias in the present work. 

### Enzyme activities

**Figure 4:**
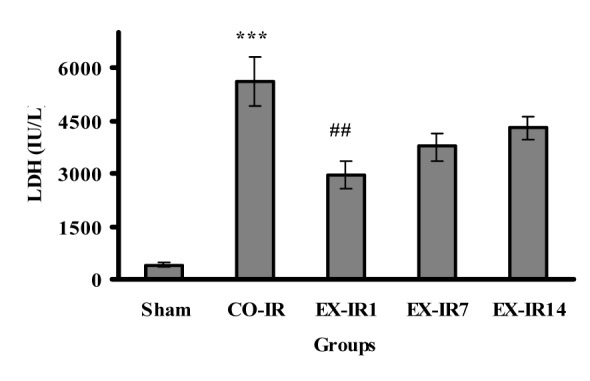
Serum lactate dehydrogenase (LDH) activity at the end of reperfusion. CO-IR, control + ischemia–reperfusion; EX-IR1, exercise + IR performed after 1 day of rest; EX-IR7, exercise + IR performed after 7 days of rest; EX-IR14; exercise + IR performed after 14 days of rest. ***P < 0.001 vs sham and ^##^P < 0.001 vs CO-IR

As seen in Figure 4(Fig. 4), LDH activities in the serum collected at the end of reperfusion was increased to 5618 ± 690 U/L in the CO-IR group. The LDH elevations was decreased by 48 % at day one and 32 % at day 7 post exercise, compared to those in the CO-IR group, respectively.

**Figure 5:**
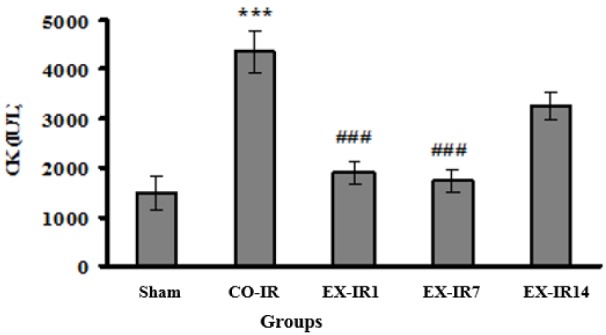
Serum creatine kinase (CK) activity at the end of reperfusion. CO-IR, control + ischemia–reperfusion; EX-IR1, exercise + IR performed after 1 day of rest; EX-IR7, exercise + IR performed after 7 days of rest; EX-IR14; exercise + IR performed after 14 days of rest. ***P < 0.001 vs sham and ^###^P < 0.0001 vs CO-IR

As seen in Figure 5(Fig. 5), CK activities was increased to 4350 ± 424 U/L in the CO-IR group. The CK elevations were decreased by 56 % at day one and 60 % at day 7 post exercise, compared to those in the CO-IR group.

## Discussion

Reduction of myocardial damage during IR injury can be accounted as a major importance in decreasing the mortality and morbidity of coronary heart disease (Michelsen et al., 2012[21]). The results of the present study show that short-term HIIT protects the heart against an* in vivo* IR injury as evidenced by significant reduction in infarct size and serum activities of CK and LDH. However, the present data fail to support the notion that HIIT attenuates ischemic-induced arrhythmias. The data also indicate that cardioprotection induced by HIIT remained for at least 7 days following detraining. 

The effects of exercise intensity in providing cardioprotection in an ischemic insult showed that exercise training at an intensity of 55-60 % VO_2_max, 40 min per day is below the threshold intensity necessary to improve cardiac tolerance against IR injury (Starnes et al., 2005[31]). However, at the same time, Lennon et al. (2004[19]) reported that 60 min/day treadmill exercise both at a moderate (55 % VO_2_max) and at a high intensity (75 % VO_2_max) provides equivalent protection against IR injury. What differs most in these two researches is the duration of exercise. Therefore, two important factors in EICP to focus upon are intensity and duration of exercise (Kavazis, 2009[17]). However, the most important variable in determining cardioprotection is the exercise intensity (Rankin et al., 2012[28]). In addition, cardioprotection induced by high intensity training seems to be better and superior (Gibala et al., 2012[11], 2006[12]; Haram et al., 2009[14]; Tjønna et al., 2008[35]; Freyssin et al., 2012[9]; Guiraud et al., 2012[13]; Wisløff et al., 2007[39], 2009[37]). The improvement of VO_2_peak is greater with high-intensity compared to low-intensity exercise (Jensen et al., 1996[16]; Wisløff et al., 2007[39]). Further, the high-intensity sprint training protects the heart against IR-induced diastolic dysfunction (Libonati et al., 2005[20]). According to these results, in the present study the high intensity exercise was chosen. Our data showed that HIIT for 30 min per day can protect the heart against IR injury and decreases infarct size nearly by 50 % compared to sedentary group. This finding agrees with previous studies using both *in vivo *and *in vitro* IR models to study EICP (Calvert and Lefer 2013[5]; Zhang et al., 2007[40]; Lennon et al., 2004[19]). Regarding to long-term physical activity, Bowles et al. (1992[2]) reported that coronary flow during early reperfusion after global ischemia in the isolated rat heart was greatest in animals that had high-intensity treadmill running compared with a lower intensity regimen for 11-18 wk. Another study also showed that high intensity treadmill running as 10 × 4-min bouts for 12 wk improved arterial function and this is maintained in small arteries even when exposed to ischemia and reperfusion (Symons et al., 2003[33]). Although the short-term exercise did not induce any structural changes in the rat heart, molecular adaptive changes did occur and may have a significant impact in attenuating myocardial injury (Dayan et al., 2005[6]). Our study showed that short-term exercise provided myocardial protection against IR injury by decreasing IS, confirming the results of previous studies.

Our results show that short-term training did not prevent IR-induced arrhythmias which are in agreement to findings of Quindry et al. (2012[27]) that indicated no arrhythmia differences existed between exercise and sedentary groups after 3 consecutive days of continuous treadmill running during 50 min ischemia. However, these researchers previously observed antiarrhythmic protection in exercised hearts during 20 min ischemic insult. This issue confirms time-dependent nature of IR injury and occurrence of tissue death after 20 min of ischemia (Powers et al., 2008[24]). On the other hand, long-term (6 weeks) voluntary exercise confers resistance to cardiac arrhythmias that is in contrast to our results. This type of exercise can lead to reduced basal heart rate due to a reduction in cardiac sympathetic activity and an increase in vagal activity or both (Beig et al., 2011[1]). The reasons for this discrepancy may be due to differences in the type of the training protocol, voluntary versus forced exercise, as well as duration of the exercise.

In conjunction with how long the heart maintains its protective effect following cessation of exercise, Lennon et al. reported that cardioprotective effect of continuous exercise (3 sessions) sustained at 1, 3, and at least 9 days post exercise and disappeared after 18 days (Lennon et al., 2004[19]). Moreover, following 4 weeks of voluntary exercise with ~7.4 ± 0.2 km/d intensity, EICP was sustained for a week after cessation of training (Calvert et al., 2011[4]). In another study, Moholdt et al. (2009[23]) reported that 4 weeks of HIIT (at 90 % of maximum heart rate) or CET (at 70 % of maximum heart rate) increased VO_2_peak in CABG patients, which persisted for six months post HIIT period by exercising moderate to high intensity at home. In our experiment, the cardioprotective effect of HIIT remained at least up 7 days after exercise cessation. Increased levels of LDH and CK during IR injury, as biochemical markers of tissue damage, were attenuated by HIIT at 1 and 7 days post exercise. 

Although extrapolation of findings from experimental animals to humans should be done cautiously, our data suggest that high intensity interval treadmill-running even for short-term can have the protective effect and improve heart tolerance against ischemia and reperfusion in rat. Importantly, these improvements are persisted at least 7 days post exercise. 

## Acknowledgements

This work was supported by Exercise Physiology Research Center, Baqiyatallah University of Medical Sciences, Tehran, IR Iran with grant NO 340/14/6-3-220. 

## Conflict of interest

The authors declare that they have no conflict of interest.

## Figures and Tables

**Table 1:**
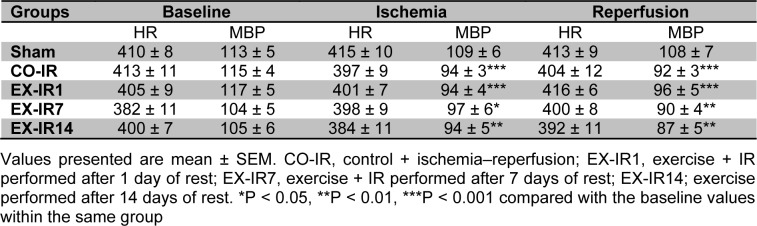
Hemodynamics parameters in experimental groups

**Table 2:**
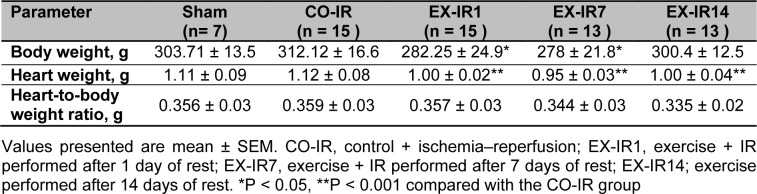
Post exercise animal body and heart weights, and heart to body weight ratio in all groups
